# Elevated Serum Amyloid a Levels Are not Specific for Sarcoidosis but Associate with a Fibrotic Pulmonary Phenotype

**DOI:** 10.3390/cells10030585

**Published:** 2021-03-07

**Authors:** Els Beijer, Claudia Roodenburg-Benschop, Milou C. Schimmelpennink, Jan C. Grutters, Bob Meek, Marcel Veltkamp

**Affiliations:** 1 Interstitial Lung Diseases Centre of Excellence, Department of Pulmonology, St. Antonius Hospital, 3435 CM Nieuwegein, The Netherlands; e.beijer@antoniusziekenhuis.nl (E.B.); c.benschop@antoniusziekenhuis.nl (C.R.-B.); m.schimmelpennink@antoniusziekenhuis.nl (M.C.S.); j.grutters@antoniusziekenhuis.nl (J.C.G.); 2 Department of Medical Microbiology and Immunology, St. Antonius Hospital, 3435 CM Nieuwegein, The Netherlands; b.meek@antoniusziekenhuis.nl; 3 Division of Heart and Lungs, University Medical Centre, 3584 CX Utrecht, The Netherlands; 4 ILD Research, Koekoekslaan 1, 3435 CM Nieuwegein, The Netherlands

**Keywords:** sarcoidosis, SAA, fibrosis

## Abstract

Elevated Serum Amyloid A (SAA) levels have been found in several inflammatory diseases, including sarcoidosis. SAA is suggested to be involved in sarcoidosis pathogenesis by involvement in granuloma formation and maintenance. We hypothesized that SAA serum levels would be higher in sarcoidosis compared to other non-infectious granulomatous and non-granulomatous diseases. SAA levels were measured in serum from sarcoidosis, Hypersensitivity pneumonitis (HP), and (eosinophilic) granulomatosis with polyangiitis ((E)GPA) patients. Idiopathic pulmonary fibrosis (IPF) patients were included as non-granulomatous disease group. SAA levels of patients with sarcoidosis (31.0 µg/mL), HP (23.4 µg/mL), (E)GPA (36.9 µg/mL), and IPF (22.1 µg/mL) were all higher than SAA levels of healthy controls (10.1 µg/mL). SAA levels did not differ between the diagnostic groups. When SAA serum levels were analyzed in sarcoidosis subgroups, fibrotic sarcoidosis patients showed higher SAA levels than sarcoidosis patients without fibrosis (47.8 µg/mL vs. 29.4 µg/mL, *p* = 0.005). To conclude, the observation that fibrotic sarcoidosis patients have higher SAA levels, together with our finding that SAA levels were also increased in IPF patients, suggests that SAA may next to granulomatous processes also reflect the process of fibrogenesis. Further studies should clarify the exact role of SAA in fibrosis and the underlying mechanisms involved.

## 1. Introduction

Serum amyloid A (SAA) is produced by the liver during the acute phase reaction (APR) which serves to eliminate foreign pathogens in a way that has not fully been elucidated yet [1]. Elevated SAA levels have also been found in several inflammatory diseases, including rheumatoid arthritis, diabetes type II, Crohn’s disease [2], and sarcoidosis [3,4,5]. Sarcoidosis is a systemic disease which is characterized by formation of non-caseating granulomas through a complex immunological process. Lymph nodes, skin, eyes, and lungs are the most frequent involved organs [6]. The etiology of the disease is currently not completely clear, but probably results from the complex interplay between different etiologic agents and the immune system in predisposed individuals [7].

Multiple effects of SAA may relate to its contribution in sarcoidosis pathogenesis. First, SAA was found to contribute to Th17-cell proliferation and Th17 cytokine production [8]. Th17-cells have been found to contribute in sarcoidosis pathogenesis [9,10] and in granuloma formation [11,12]. Second, Chen et al. [13] studied the role of SAA in the regulation of granulomatous inflammation in sarcoidosis and found significant presence of SAA in granulomas of sarcoidosis patients which exceeded the intensity of SAA staining found in tissue from patients with tuberculosis, granulomatous polyangiitis, or hypersensitivity pneumonitis [13]. They, therefore, proposed that SAA might contribute to granuloma formation in sarcoidosis due to SAA overproduction as result of the intensity of the immune response in this disease. This accumulation of SAA might result in misfolding or aggregation of this protein and formation of complexes with other matrix proteins, leading to granuloma formation and entrapment of microbial antigens or autoantigens [13].

In our previous work we identified trigger-related phenotypes in sarcoidosis based on immunological sensitization for mycobacterial antigens and presence of Cutibacterial antigens in granulomas [14,15]. Based on its function as an acute phase protein upregulated by infections and contribution in bacterial clearance [1,16,17], together with its considered role as a biomarker in sarcoidosis, increased SAA levels may reflect underlying microbial antigens or autoantigens as triggers in a subgroup of sarcoidosis patients. This prompted us to further investigate the role of SAA in this heterogeneous disease. Based on the study of Chen et al. [13], we hypothesize that SAA serum levels will be higher in sarcoidosis compared to other non-infectious granulomatous and non-granulomatous diseases. Furthermore, we hypothesize that SAA levels in sarcoidosis patients with immunological sensitization for bacterial and autoantigens will be higher compared to patients with immunological sensitization for inorganic triggers like metals or silica.

## 2. Material and Methods

### 2.1. Study Population

Two sarcoidosis cohorts previously studied in the St. Antonius hospital were included in the study [14,18]. Sarcoidosis was diagnosed according to the criteria of the American Thoracic Society/European Respiratory Society [6]. Hypersensitivity pneumonitis (HP) and (eosinophilic) granulomatosis with polyangiitis ((E)GPA) patients participating in our biobank study were included as granulomatous disease groups other than sarcoidosis. A cohort of patients with IPF [19] were included as non-granulomatous disease group. SAA levels of the granulomatous disease and non-granulomatous disease groups were compared to a healthy control group.

Written consent was obtained from all patients and healthy controls. The study was approved by the Medical research Ethics Committees United (MEC-U) of the Antonius hospital (R05-08A).

### 2.2. SAA Serum Measurements and Correlations

SAA levels were determined in serum from patients and controls through a SAA human ELISA kit (KHA0012, Invitrogen, Camarillo, CA, USA) following the manufacture’s protocol. Data of C-reactive protein (CRP) and lung function at the time of serum collection for SAA measurements were retrospectively collected from medical records. Correlations between SAA and CRP and lung function were determined for patients of whom data were available at the time of serum collection used for SAA measurements.

### 2.3. Sarcoidosis Patients with Immunological Sensitization to Either Bacterial, Inorganic or Autoantigens

Immunological sensitization to mycobacterial antigens, *Cutibacterium acnes* (*C. acnes*) catalase, vimentin, metals, and silica were previously determined [14]. Sarcoidosis patients in which sensitization was determined were divided in the following sub-groups: bacterial sensitization (sensitization to mycobacterial antigens or *C. acnes* catalase), auto-antigen sensitization (sensitization to vimentin), and inorganic sensitization (sensitization to aluminum, beryllium, zirconium, or silica). SAA serum levels of sarcoidosis patients with immunological sensitization to bacterial and autoantigens were compared to the SAA levels of sarcoidosis patients with immunological sensitization to inorganic antigens.

### 2.4. Statistics

Study data were collected and managed using REDCap electronic data capture tools hosted at St. Antonius hospital. Data were analyzed using IBM SPSS statistics version 24. An unpaired T-test was used to compare numerical data between the groups. Non-parametric tests were used for non-normally distributed data (Mann–Whitney U test). Categorical data were compared between the groups using the Chi-squared test. If expected cell frequencies were below 5, Fisher’s exact test was used for categorical data up to two categories. Correlation between SAA and other variables was determined with Spearman’s rho correlation. *p*-values < 0.05 were considered significant.

## 3. Results

### 3.1. Demographics of Study Patients

SAA serum levels were measured in 215 sarcoidosis patients, 30 HP patients, 11 (E)GPA patients, 68 IPF patients, and 200 healthy controls. Demographics can be found in Table 1.

### 3.2. SAA Serum Levels Do Not Differ between Patients with Granulomatous and Non-GranulomAtous Diseases but Are Higher in General Compared to Healthy Controls

**Figure 1:**
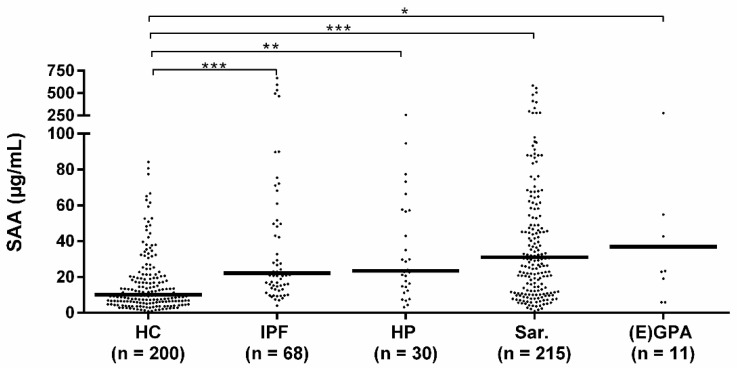
Serum amyloid A (SAA) levels in patients and healthy controls. Median SAA levels of idiopathic pulmonary fibrosis (IPF) (22.1 IQR 55.5), HP (23.4 IQR 43.4), sarcoidosis (31.0 IQR 56.3) and (E) GPA patients (36.9 IQR 123.7) were all higher than the median SAA level of healthy controls (10.1 IQR 15.6). Median SAA levels did not significantly differ between the diagnostic groups. HC: healthy control, Sar: sarcoidosis, HP: hypersensitivity pneumonitis, (E) GPA: (eosinophilic) granulomatosis with polyangiitis ((E) GPA), IPF: idiopathic pulmonary fibrosis. *, **, *** *p*-values were calculated using Kruskal–Wallis test including pairwise comparisons.

Median SAA levels of sarcoidosis, HP, (E)GPA, and IPF patients were all higher than the median SAA level of healthy controls. No differences in median SAA levels were observed between the different patient groups (Figure 1). When comparing the granulomatous diseases (sarcoidosis, HP, EAA, (E) GPA) with the non-granulomatous disease patients (IPF), no difference in median SAA levels was observed either (median 30.5 IQR 55.3 vs. 22.1 IQR 55.5, *p* = 0.737).

### 3.3. SAA Correlates with CRP, Lung Function and Use of Immunosuppressive Medication

Significant moderate positive correlations were found between SAA and CRP serum levels. Significant weak negative correlations were observed between SAA and FEV1 percentage predicted (Table 2). When analyzing the correlations per diagnostic group, only in sarcoidosis and IPF patients the correlation between SAA and CRP remained significant (R = 0.384 and R = 0.598, respectively, both *p* < 0.001). FEV1 showed only a weak negative correlation with SAA in sarcoidosis patients (R = −0.191, *p* = 0.012). Although no overall correlation was observed between SAA and DLCO, the sub analysis revealed a weak negative correlation in sarcoidosis patients (R = −0.223, *p* = 0.005) and a trend in IPF patients (R = −0.265, *p* = 0.050).

### 3.4. SAA Levels Are Higher in Patients Using Immunosuppressive Medication

As a part of the patients with sarcoidosis, HP, and (E)GPA was using medication at the time of SAA determination, we analyzed whether SAA levels differed between patients with and without medication. Overall, patients using immunosuppressive medication showed higher SAA levels than patients without medication. When considering the type of medication used, we only observed significant higher SAA levels in patients using prednisone (Table 3). When we compared the different diagnostic groups for SAA levels and the use of medication, we only observed significantly higher median SAA levels in sarcoidosis patients with medication compared to sarcoidosis patients without medication (44.4 µg/mL vs. 26.3 µg/mL, *p* = 0.001). When we compared SAA levels per type of medication used, we again found significant higher SAA levels in sarcoidosis patients using prednisone. No difference was observed for the other types of medication used or for CRP levels.

### 3.5. SAA Levels Do Not Differ Between Sarcoidosis Patients with Immunological Sensitization to Bacterial and Autoantigens or Inorganic Antigens

**Figure 2:**
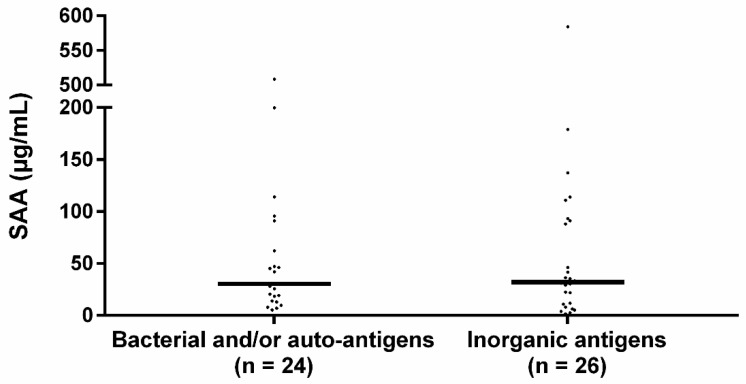
Serum SAA levels in sensitized sarcoidosis sub-groups. Median SAA levels between bacterial sensitized and/or autoantigen sensitized and inorganic agent sensitized sarcoidosis patients did not significantly differ. The percentage of patients using immunosuppressive medication did not significantly differ between the bacterial and/or autoantigen and inorganic sensitized group (33.3% vs. 46.2% respectively, *p* = 0.355) nor did the percentage of patients using prednisone (20.8% vs. 42.3%, *p* = 0.104). Bacterial: sensitization to mycobacterial antigens or *C. acnes* catalase, autoantigen: sensitization to vimentin, inorganic: sensitization to aluminum, beryllium, zirconium, or silica. *p*-values were calculated using Mann–Whitney U test.

The median SAA level of patients sensitized to bacterial and/or autoantigens was 30.4 IQR 68.7 and to inorganic antigens 32.1 IQR 81.6 (Figure 2). SAA levels did not significantly differ between the groups (*p* = 0.719).

### 3.6. SAA Serum Levels Are Higher in Sarcoidosis Patients with Parenchymal Fibrosis

Median SAA levels were compared between sarcoidosis patients with different Scadding stages. Comparing SAA levels between all Scadding stages showed significant differences in median SAA levels between the five Scadding stages (*p* = 0.025, Table 4). Pairwise comparisons showed that Scadding stage IV patients had significantly higher median SAA levels compared to Scadding stage III patients (adj. *p* = 0.012). Furthermore, when SAA serum levels of Scadding stage IV (fibrosis) patients were compared with all other Scadding stages together (no fibrosis), significant higher median SAA levels were observed in the fibrotic group (*p* = 0.005). When we only examined sarcoidosis patients without use of immunosuppressive medication, median SAA levels were still significantly higher in the fibrotic group (48.9 vs. 22.3, *p* = 0.020). No differences in median CRP levels were found between any of the Scadding stages.

## 4. Discussion

Although previous studies have suggested a possible role for SAA in the pathogenesis of sarcoidosis, our study shows that this is not reflected by differences in serum levels between sarcoidosis and other pulmonary (granulomatous) diseases. SAA levels were elevated in patients with sarcoidosis, HP, (E)GPA, and IPF compared to healthy controls. Within the group of sarcoidosis patients, SAA levels did not differ between patients with immunological sensitization to either bacterial and autoantigens, or inorganic antigens. The important findings from our study are the increased SAA levels in patients with IPF compared to healthy controls and the increased SAA levels in sarcoidosis patients with pulmonary fibrosis compared to sarcoidosis patients without parenchymal fibrosis.

Even though in vitro activated monocyte/macrophages are capable of producing SAA [20] and sarcoidosis is a monocyte/macrophage driven disease [21], this did not result in an overall increase in circulating SAA when compared to other inflammatory diseases. It may be that SAA produced in tissue does not necessarily contribute to the circulatory levels of SAA and vice versa. For instance, SAA was not observed in granulomas of GPA patients, while expression and serum levels were increased [13,22]. Likewise, we observed elevated SAA levels in serum of HP patients while results of Chen et al. demonstrated limited to no SAA staining in granulomas of HP patients [13].

Chen et al. did observe that SAA patterns within granulomas of sarcoidosis patients were different from patients with other granulomatous diseases including chronic beryllium disease (CBD) and Crohn’s disease. However, a lymph node of a patient infected with *Mycobacteria Tuberculosis* demonstrated patchy expression of SAA, localized mostly around the periphery of necrotizing granulomas [13]. For this reason, combined with the fact that SAA is an acute phase protein upregulated by infections [1] and may have a role in clearance of bacterial antigens [16,17], we hypothesized that sarcoidosis patients sensitized to bacterial antigens had higher SAA levels than sarcoidosis patients sensitized to inorganic antigens such as metals and silica. The results of our study do not support this hypothesis. It is possible that blood is not the correct compartment to answer this question, and that immunohistochemical staining of SAA in granulomas may be required to determine whether there are different patterns between those subgroups of sarcoidosis patients and between sarcoidosis and other granulomatous disease patients.

Interestingly, we observed that sarcoidosis patients with a pulmonary fibrotic phenotype had higher serum SAA levels than sarcoidosis patients without pulmonary fibrosis. Chen et al. showed that the extent of SAA staining correlated with the degree of collagen deposition [13]. This suggests that SAA serum levels and tissue expression is associated with chronic inflammation and fibrosis. In line with the association between SAA and fibrosis, we observed a correlation between SAA and DLCO % predicted in sarcoidosis. In patients with systemic sclerosis (SSc), SAA was found to associate with pulmonary involvement and a negative correlation between SAA and DLCO was observed as well. Moreover, within SSc patients with different HRCT patterns, SSc patients with reticulation or honeycombing on HRCT, indicative for pulmonary fibrosis, had the highest serum SAA levels [23].

Comparable to our results, Vietri et al. showed that the SAA levels in serum were higher in IPF patients than in healthy controls [24]. They, however, also observed that IPF patients had significantly higher SAA levels than other ILD groups including sarcoidosis. This is not in line with our results. Although Vietri et al. included a group of chronic sarcoidosis patients, it is not described whether these patients were fibrotic or not. Since we observed higher SAA levels among fibrotic sarcoidosis patients compared to non-fibrotic sarcoidosis patients, such a difference in the sarcoidosis cohort may explain the discrepancy in the observations of Vietri et al. and ours.

In a previous study Vietri et al. [25] hypothesized that SAA is overproduced in response to fibrotic and hypoxic stimuli, since increased SAA levels are also observed in chronic obstructive pulmonary disease (COPD) [26] and obstructive sleep apnea (OSA) [27]. Another possible explanation for the association between increased SAA levels and fibrosis may be that fibrosis is often accompanied by low-grade inflammation and tissue-barrier disruption [28]. Since SAA contributes to (bacterial) antigen clearance, its production may be upregulated in response to the barrier disruption of fibrotic areas. A distinct, contrasting explanation is perhaps an anti-fibrogenic effect of SAA. This was observed in a mouse model of CCl_4_-induced hepatic fibrogenesis in which liver fibrosis was enhanced after treatment with anti-SAA antibodies [29]. Hypothetically, SAA production may be induced in response to fibrogenesis, to prevent further fibrosis formation. We observed an overall correlation between CRP and SAA. After performing an analysis in the diagnostic groups, we only observed a significant correlation in sarcoidosis and IPF patients, probably explained by the larger sample size of these patient groups. Although acute phase proteins such as CRP and SAA correlate quite well in several diseases [30,31,32], in our study SAA levels are higher in sarcoidosis patients with a pulmonary fibrotic phenotype compared to sarcoidosis patients without fibrosis, while CRP levels do not differ between these groups. This observation shows that determining SAA levels next to CRP levels can be meaningful in sarcoidosis patients, since those two acute phase proteins reflect different phases of disease.

An intriguing observation was the higher SAA level in patients using immunosuppressive medication at the time of serum collection for SAA measurement. This effect was specifically seen in sarcoidosis patients using prednisone. Comparable to our observation, Yamada et al. also observed higher SAA levels in rheumatoid arthritis patients receiving oral prednisolone [33]. An inducing effect of prednisolone on SAA production of HepG2 cells has been found [34]. Interestingly, a major increase in SAA expression in human monocytes and macrophages was found after a combined treatment with lipopolysaccharide (LPS) and dexamethasone or methylprednisolone [20]. In a previous study, we showed the presence of *C. acnes* lipoteichoic acid (LTA) in granulomas of sarcoidosis patients [15]. Comparable to LPS of gram-negative bacteria, LTA is a cell wall polymer of gram-positive bacteria and plays a role in bacterial growth, membrane homeostasis, and virulence [35]. Furthermore, LTAs have shown to be immunogenic and activate the innate immune system via TLR2 and NLRP6 [36,37]. It is possible that bacterial molecules including LPS and LTA together with glucocorticoids stimulate macrophages within granulomas to produce SAA, explaining the higher SAA levels observed in sarcoidosis patients using prednisone.

Based on the results of our study, it is not possible to determine whether medication itself has an effect on SAA levels or that SAA levels are increased in the more severe patient group needing immunosuppressive therapy. However, since we observed higher SAA levels in fibrotic sarcoidosis patients who did not use medication, there have to be other factors that determine the higher SAA levels we observed in fibrotic sarcoidosis patients compared to non-fibrotic sarcoidosis patients. It has been suggested that SAA is useful as a biomarker in sarcoidosis, since it is not dramatically affected by immunosuppressive therapy [38]. Further studies will have to clarify the association between immunosuppressive therapy and SAA, for example by performing SAA measurements before, during, and after treatment.

Our study has some limitations. First, SAA levels were not measured at the time of diagnosis for all patients. For this reason, we were not able to analyze whether increased SAA levels were predictive for the disease course. We were not able to measure SAA levels of all patients at the time of diagnosis since we made use of existing patient cohorts from which serum at the time of diagnosis was not available. However, by making use of these patient cohorts, we were able to analyze SAA levels between sensitization subgroups within the sarcoidosis patients, which had never been performed before. A second limitation was that a proportion of the patients were using immunosuppressive medication at the time of SAA measurements. We were not able to track down whether increased SAA levels observed in the patients using medication was due to the mediation itself or rather due to the more severe disease course of these patients. Further studies will have to reveal the effect of immunosuppressive medication on the disease course, for instance by measuring SAA levels at diagnosis and during follow-up in one cohort of patients.

**Figure 3:**
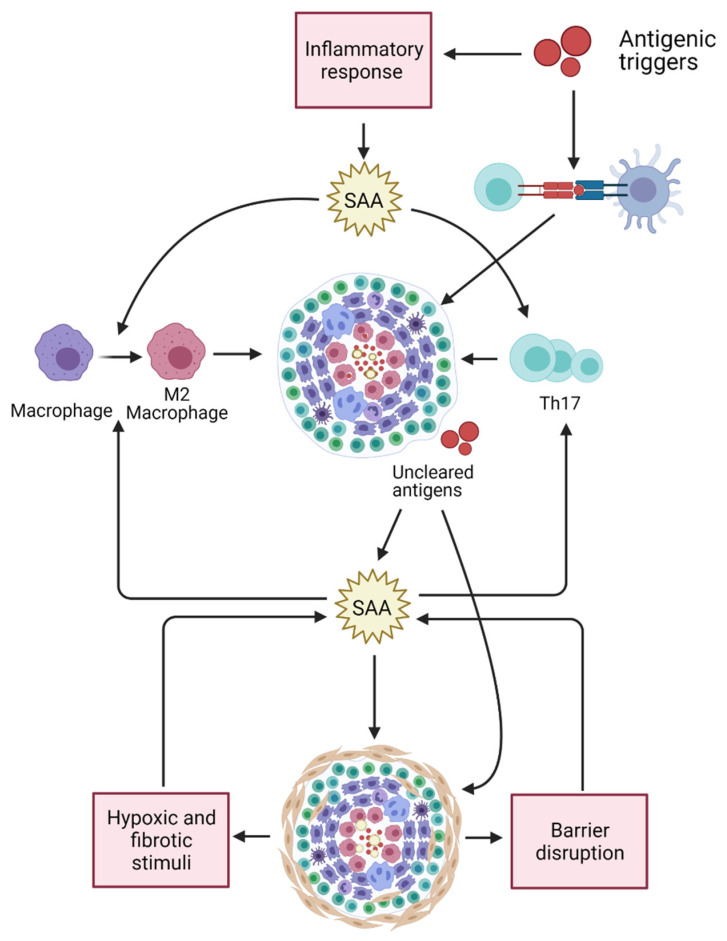
Multiple effects of SAA may relate to its contribution in sarcoidosis pathogenesis. SAA levels are upregulated in response to (bacterial) antigens stimulating the innate immune system. SAA contributes in Th17-cell proliferation, Th17 cytokine production, and in M2 polarization of macrophages. Both Th17 cells as well as M2 macrophages are thought to be involved in sarcoidosis pathogenesis and granuloma formation. Accumulation of SAA might result in misfolding or aggregation of SAA and formation of complexes with other matrix proteins, leading to granuloma formation and entrapment of antigens. SAA serum levels and tissue expression may result in chronic inflammation and fibrosis. SAA levels may be further increase in response to fibrotic and hypoxic stimuli. Fibrosis is often accompanied by low-grade inflammation and tissue-barrier disruption, which may also lead to an upregulation of SAA levels since SAA contributes to (bacterial) antigen clearance. Created with BioRender.com (accessed on 3 March 2021).

To conclude, increased SAA levels in serum is not specifically associated with sarcoidosis but can also be found in other granulomatous diseases such as HP and (E)GPA. In patients with sarcoidosis, the level of serum SAA is associated with the concomitant use of glucocorticoids. The fact that elevated SAA levels were observed in patients with IPF and were significantly higher in sarcoidosis patients with pulmonary fibrosis compared to patients without parenchymal fibrosis, suggests that SAA may also reflect the process of fibrogenesis. Further studies should clarify the exact role of SAA in fibrosis and the underlying mechanisms involved in the effects of glucocorticoids on SAA levels in sarcoidosis. Figure 3 provides a schematic presentation of multiple effects by which SAA may contribute in sarcoidosis pathogenesis.

## Figures and Tables

**Table 1:** Demographics of study patients.

	HC (*n* = 200)	Sar (*n* = 215)	HP (*n* = 30)	(E)GPA (*n* = 11)	IPF (*n* = 68)
Age ^a^	46.6 ± 9.2	48.2 ± 11.8	59.6 ± 9.5	54.7 ± 13.9	69.5 ± 8.9
Sex (male)	92 (46)	117 (54)	15 (50)	7 (64)	60 (88)
Ethnicity (White)		190 (89)	30 (100)	11 (100)	67 (99)
Smoker (Never)		105 (49)	13 (43)	4 (36)	16 (24)
Immunosuppressive medication	0	101 (47)	12 (40)	6 (55)	0
CRP (mg/L)		2.0 IQR 4.0	2.0 IQR 4.0	2.0 IQR 23.0	3.5 IQR 5.0
FVC % predicted		94.3 ± 20.3	78.5 ± 21.7	90.0 ± 21.2	80.1 ± 20.1
FEV1 % predicted		84.9 ± 21.5	81.2 ± 22.0	86.2 ± 20.1	82.9 ± 18.3
DLCO % predicted		73.5 ± 17.4	46.8 ± 13.8	58.9 ± 20.2	41.5 ± 11.4
Scadding stage			NA	NA	NA
0		44 (21)			
I		45 (21)			
II		50 (23)			
III		32 (15)			
IV		40 (19)			
Unknown		4 (2)			

Values are shown as absolute numbers with percentages in brackets; ^a^ Age, FVC % predicted, FEV1 % predicted and DLCO % predicted are shown as mean ± std. CRP, is shown as median IQR.; Medication consisted of immunosuppressive medication including prednisone, methotrexate, azathioprine, infliximab, leflunomide, adalumimab, hydroxychloroquine and cellcept.; CRP was available from 207 sarcoidosis patients, 27 HP patients, 9 (E)GPA patients and 68 IPF patients.; FVC was available from 166 sarcoidosis patients, 27 HP patients, 11 (E)GPA patients and 59 IPF patients; FEV1 was available from 174 sarcoidosis patients, 26 HP patients, 11 (E)GPA patients and 60 IPF patents; DLCO was available from 160 sarcoidosis patients, 25 HP patients, 7 (E)GPA patients and 55 IPF patients; HC: healthy control, Sar: sarcoidosis, HP: hypersensitivity pneumonitis, (E)GPA: (eosinophilic) granulomatosis with polyangiitis ((E)GPA), IPF: idiopathic pulmonary fibrosis, CRP: C-reactive protein, FVC: Forced vital capacity, FEV1: Forced expiratory volume in 1 s, DLCO: Diffusing capacity.

**Table 2:** Spearman’s rho correlations with SAA.

	R	*p*
CRP (mg/L) (*n* = 311)	0.408	<0.001
FVC % predicted (*n* = 263)	−0.084	0.173
FEV1 % predicted (*n* = 271)	−0.154	0.011
DLCO % predicted (*n* = 245)	−0.111	0.082

Correlations and *p*-values were determined by Spearmans’s rho; CRP: C-reactive protein, FVC: Forced vital capacity, FEV1: Forced expiratory volume in 1 s, DLCO: Diffusing capacity.

**Table 3:** Median SAA levels in Sarcoidosis, HP, and (E)GPA patients with and without immunosuppressive medication.

Use of Immunosuppressive Mediation	No (*n* = 404)	Yes (*n* = 119)	*p*
SAA (µg/mL)	16.9 IQR 30.2	43.8 IQR 72.5	<0.001
Prednisone	No (*n* = 430)	Yes (*n* = 93)	
SAA (µg/mL)	16.9 IQR 30.7	49.0 IQR 93.10	<0.001
MTX	No (*n* = 488)	Yes (*n* = 35)	
SAA (µg/mL)	20.6 IQR 40.0	25.9 IQR 56.4	0.536
Aza	No (*n* = 515)	Yes (*n* = 8)	
SAA (µg/mL)	20.6 IQR 40.0	32.2 IQR 32.7	0.232
Inf	No (*n* = 516)	Yes (*n* = 7)	
SAA (µg/mL)	20.7 IQR 40.0	35.0 IQR 87.6	0.531

MTX: Methotrexate, Aza: Azathioprine, Inf: infliximab. Sub-analyses were performed only for medications used by 5 or more patients. Leflunomide and hydroxychloroquine was used by 1 patient and adalimumab and cellcept by 2 patients.

**Table 4:** SAA levels among sarcoidosis patients with different Scadding stages.

	Scadding Stage					*p*
	0 (*n* = 44)	I (*n* = 45)	II (*n* = 50)	III (*n* = 32)	IV (*n* = 40)	
SAA (µg/mL)	27.8 IQR 43.5	32.7 IQR 67.7	31.3 IQR 48.7	21.6 IQR 39.1	47.8 IQR 76.4	0.025
CRP (mg/L)	2.0 IQR 4.0	3.0 IQR 7.0	3.0 IQR 4.0	2.0 IQR 6.0	2.5 IQR 5.0	0.382
	0, I, II, III				IV	
SAA (µg/mL)	29.4 IQR 51.3				47.8 IQR 76.4	0.005
CRP (mg/L)	2.0 IQR 5.0				2.5 IQR 5.0	0.715

Values are shown as median IQR. CRP was missing from 2 Scadding stage II patients, 3 Scadding stage III patients and 2 Scadding stage IV patients; *p*-values for multiple comparisons were calculated using Kruskal-Wallis test. Multiple comparisons were adjusted by the Bonferroni correction. *p*-values comparing 2 groups were defined by the Mann-Whitney U test.

## Data Availability

The data presented in this study are available on request from the corresponding author.
